# Spontaneous pneumomediastinum: diagnostic and therapeutic interventions

**DOI:** 10.1186/1749-8090-3-59

**Published:** 2008-11-03

**Authors:** Faisal Al-Mufarrej, Jehangir Badar, Farid Gharagozloo, Barbara Tempesta, Eric Strother, Marc Margolis

**Affiliations:** 1 The George Washington University Medical Center, Department of Surgery, 2300 Eye Street NW, Washington, DC 20037, USA; 2 Washington Institute of Thoracic and Cardiovascular Surgery, 2175 K Street NW, Washington DC 20037, USA

## Abstract

**Objectives:**

The objective of this case series is to review our experience with spontaneous pneumomediastinum, review the available literature, and refine the current clinical approach to this uncommon condition.

**Methods:**

The case notes of all patients admitted to the George Washington University Medical Center with spontaneous pneumomediastinum from April 2005 to June 2008 were retrospectively reviewed, indentifying seventeen patients on whom various data was collected and analyzed.

**Results:**

The typical patient is a young man. The commonest presenting complaint is chest pain. Odynophagia and subcutaneous emphysema are common. Leucocytosis is uncommon. The need for swallow studies, antibiotics, and prolonged hospitalization is uncommon. Most patients have no recurrences or sequelae on long-term follow-up.

**Conclusion:**

Spontaneous pneumomediastinum is an uncommon, self-limiting condition. Due to concerns for the integrity of the aero-digestive tract, the finding of spontaneous pneumomediastinum usually results in unnecessary radiological investigations, dietary restriction and antibiotic administration with prolonged hospitalization.

## Introduction

Pneumomediastinum or air in the mediastinum may originate from the esophagus, lungs, or bronchial tree. As suggested by a handful of small case series in the literature [1-4], spontaneous pneumomediastinum is an uncommon, self-limiting condition. It results from alveolar rupture-otherwise known as the Macklin phenomenon. Alveolar rupture results from high intra-alveolar pressures, low peri-vascular pressures, or both. Air escaping from the alveoli tracks into the mediastinum during the breathing cycle as the pressure in the mediastinum decreases relative the pulmonary parenchymal pressure. From there, air may track into the cervical subcutaneous tissues, epidural space [5], pericardium [6], and/or peritoneal cavity [7,8].

Spontaneous pneumomediastinum usually results from bronchial hyper-reactivity or barotraumas. Bronchial constriction may be due to asthma or inhalation [8] of toxic agents (e.g. cocaine) [9]. Barotrauma may occur with instrumentation, mechanical ventilation, or Valsalva's maneuver (expiration through resistance) that occurs with straining (e.g. during labor) or vomiting [5]. The finding of a pneumomediastinum usually places the integrity of the thoracic aero-digestive tract into question resulting in unnecessary radiological investigations, needless dietary restriction, unjustified antibiotic administration, and prolonged hospitalization.

The objective of this retrospective study is to review our experience with spontaneous pneumomediastinum, review the available literature, and refine the current clinical approach to this uncommon condition.

## Methods

We retrospectively reviewed the case notes of all patients admitted to George Washington University Hospital with pneumomediastinum from April 2005 to June 2008. All trauma patients as well as patients with intra-thoracic malignancy, hemodynamic instability, or recent aero-digestive instrumentation were excluded from the study. Seventeen patients with spontaneous pneumomediastinum were identified.

For each case, data was collected regarding demographics, likely etiology, presenting complaints, white cell count level, other laboratory abnormalities, findings on chest x-ray and computed axial tomography, results of any contrast swallow study or esophagogastroduodenoscopy done, use of antibiotics, dietary restriction, length of hospitalization, and condition on follow-up.

The Institutional Review Board of the George Washington University approved this retrospective study (IRB number 060818).

## Results

There were eleven men (65%) and six women (35%). The mean age was 25.5 years (range 19–39 years). The etiology was unclear in 29.4% of patients. Asthma and alcohol-related emesis were implicated in 23.5% of patients each, while physical activity and gastro-intestinal disease (including gastroparesis and history of ulcer disease) were seen in 11.8% of cases each. Cocaine and history of pneumothorax were noted in only 5.9% of patients each.

The commonest presenting complaint was chest pain (usually retrosternal) and dyspnea (frequency of 58.8% and 41.2%, respectively). 11.8% of patients presented with neck pain and 17.8% of them noted some degree of odynophagia. Less common symptoms included nausea/emesis (11.8%), back pain (11.8%), abdominal pain (5.9%) and fever (5.9%). None of the patients had true complaints of dysphagia. Only 11.8% had cervical subcutaneous emphysema on exam.

With the exception of one dehydrated patient with pre-renal acute renal failure and a white cell count of 19.6 × 10^9^/L, the white cell count ranged from 4.97–10.86 × 10^9^/L with an average white cell count of 9.42 × 10^9^/L. D-dimer levels were checked in all patients with chest pain and were found to be abnormal in 11.8% of cases with a range of 640–700 ng/ml.

The pneumomediastinum was visible of chest x-ray on 52.9% patients (33.3% of females and 63.6% of males), but none of the patients had any effusions noted on their chest x-rays. The pneumomediastinum resolved on chest x-ray in 2–6 days with an average of 3.5 days. Computed axial tomography (CT) of the chest was done in all patients but one. The finding of pneumomediastinum on computed axial tomography was associated with subcutaneous emphysema in 41.2%, pneumoperitoneum in 5.9% and pneumorachis in another 5.9%. A contrast swallow was done in 58.8% patients, all of which were negative. An esophagogastroduodenoscopy (EGD) with the finding of distal esophagitis was done during the same admission or soon after discharge on patients with gastrointestinal disease.

53% of cases were initially admitted to the intensive care unit for observation, while 47% were admitted to the surgical floor with 4-hourly vitals. For patients with no other co-morbidities, the length of hospital stay ranged from 23–72 hours (average of 40.5 hours). Patients admitted with significant dehydration, asthma exacerbation, or drug intoxication had longer hospital stays (5–11 days with average of 7 days).

Broad spectrum intravenous antibiotics were administered in the emergency room in 58.8% patients, 70% of whom continued to receive antibiotics during their hospitalization. The antibiotics used were variable and included various combinations of ciprofloxacin, clindamycin, moxifloxacin, piperacillin-tazobactam, cefepime, and metronidazole. 52.9% of the patients were made nil per os upon admission and advanced to regular diet in 24–48 hrs. The remaining patients were allowed to have a regular diet after a contrast swallow study had confirmed an intact esophagus.

Follow-up ranged from 6 days to 34 months with a mean follow-up of 6.72 months. All patients were asymptomatic with normal chest x-rays on follow-up.

## Discussion

Pneumomediastinum is an uncommon self-limiting benign condition that is frequently over-investigated and over-treated due to concern for missing an aero-digestive injury. A handful of small case series in the literature have suggested the benign course of this condition [1-4], but there are still not clear guidelines regarding the diagnostic and therapeutic interventions needed for patient with spontaneous pneumomediastinum. Our experience with spontaneous pneumomediastinum suggests limiting the use of swallow studies, antibiotics, and dietary restriction to allow for early discharge and better use of hospital resources.

We suggest that a complete blood count and a CT of the chest for all patients. Even for patients with obvious pneumomediastinum on chest x-ray, a CT the chest with intravenous contrast should be obtained since chest x-rays are obviously not sensitive at picking up mild disease or evaluating the chest for pathology. An elevated D-dimer may occur in patient with benign pneumomediastinum in the absence of thromboembolic disease, but leucocytosis is unusual in such patients.

Not all patients with spontaneous pneumomediastinum need contrast swallow studies. A contrast swallow study should be obtained for patients with emesis, dysphagia, gastro-intestinal disease, trauma, hemodynamic instability, fever, leucocytosis, pleural effusion, or pneumoperitoneum. While esophageal rupture (Boerhaave's syndrome) is classically described with left-sided pleural effusions, one must be suspected esophageal injury in the presence of any pleural effusion whether it is left or right-sided [10,11]. The classic Meckler's triad of esophageal rupture includes vomiting, lower chest pain, and cervical subcutaneous emphysema following overindulgence in food or alcohol. Our experience, however, demonstrates that both cervical subcutaneous emphysema and odynophagia are quite common in the setting of benign spontaneous pneumomediastinum, and that patients with either finding do not need an esophageal evaluation.

This case series clearly suggests that not all patients with emesis and pneumomediastinum (even those with pneumoperitoneum) have Boerhaave's syndrome. This is confirms the findings of a recently published small case series in the literature [12]. The mechanism of the pneumomediastinum is likely due to alveolar rupture secondary to the significant Valsalva maneuver that accompanies the vomiting reflex. The pneumomediastinum in this setting is likely benign; however, a swallow study should be done in all cases presenting with emesis. Given the gravity of delayed diagnosis of esophageal rupture, the index of suspicion must be high in cases of pneumomediastinum with the right mechanism for rupture (emesis or trauma). Esophageal rupture has also been described in patients presenting primarily with hematemesis [13]. It is important to suspect esophageal injury in such cases even in the absence of other signs of rupture (fever, leucocytosis, hemodynamic instability, pleural effusion) since early contained esophageal perforations may be subclinical and hard to recognize [13,14]. This is especially true for immunocompromised patients or ones on steroids or antibiotics.

For patients with negative swallow studies, patients can be admitted for 23 hour observation with no need to keep them nil per os. For patients who do not undergo an evaluation of their esophagus, they should be kept nil per os only overnight with advancement to regular diet the very next day as long as the white cell count remains below 11 × 10^9^/L. Intensive care is unnecessary unless patient is hemodynamically unstable or the diagnosis of esophageal injury is highly suspected. Slow diet advancement and holding up discharge until chest x-ray resolution of pneumomediastinum prolongs hospitalization with no difference in long term outcome. In addition to the contrast swallow study, patients with known gastro-intestinal disease (gastroparesis, gastro-esophageal reflux, peptic ulcer disease, etc.) who present with pneumomediastinum need an EGD in the in-patient or out-patient setting in order to evaluate the esophagus and stomach for inflammatory and ulcer disease.

There is no need for intravenous antibiotics in patients with negative swallow studies, and in patients who do not undergo an evaluation of their esophagus, antibiotics may mask an occult injury. All patients did well regardless of whether they obtained antibiotics or not, suggesting against the use of antibiotics in this patient population.

## Conclusion

Our experience with spontaneous pneumomediastinum confirmed the previously reported benign nature of this uncommon condition. While prospective studies or meta-analyses in this subject matter are clearly needed, this case series does suggest that the condition is over-investigated and over-treated. Thus, clinicians need to be more judicious with the use of hospital resources in managing patients with spontaneous pneumomediastinum.

## Competing interests

The authors declare that they have no competing interests.

## Authors' contributions

FM, JB, MM, FG, ES, and BT were all involved the care of the patients included in the study. FM, ES and BT collected the data. FM and JB did the background literature search and drafted the manuscript. MM and FG were involved in the conception of the study and the critical review of the intellectual content of the manuscript. All authors read and approved the final manuscript.
